# Green Cytoplasmic Neutrophilic Inclusion Bodies in a Patient With Aspiration Pneumonia and Bowel Perforation

**DOI:** 10.7759/cureus.41318

**Published:** 2023-07-03

**Authors:** Phillip S Wozniak, Garrett L Rampon

**Affiliations:** 1 Department of Pediatrics, Children's Mercy Kansas City, Kansas City, USA; 2 Department of Internal Medicine, University of Missouri Kansas City School of Medicine, Kansas City, USA; 3 Department of Internal Medicine, Division of Pulmonary and Critical Care Medicine, University of Missouri Kansas City School of Medicine, Kansas City, USA

**Keywords:** in-icu mortality, mortality predictors, critical illness diagnosis, blood pathology, pulmonary critical care

## Abstract

Blue-green cytoplasmic neutrophilic inclusion bodies, previously described as "green crystals of death," are a rare but likely underreported finding in critically ill patients. This finding is associated with high mortality, ranging from 31% to 100% in published case studies. These inclusion bodies have been most strongly associated with acute liver injury and lactic acidosis, but they have also been reported in critically ill patients secondary to other etiologies. Here, we report a case of blue-green neutrophilic inclusion bodies in a patient with aspiration pneumonia and severe pneumoperitoneum secondary to bowel perforation. These blue-green neutrophilic inclusion bodies offer high prognostic value for physicians, and their presence should be considered a "critical result," indicating the severity of the patient's illness.

## Introduction

Blue-green refractile neutrophilic inclusion bodies are a rare pathologic finding that has been closely associated with short-term mortality in critically ill patients [1-5]. The most common etiology associated with the development of these inclusion bodies is acute liver injury (ALI); however, this finding has also been seen in patients with *Escherichia coli* (*E. coli*) and *Klebsiella septicemia*, multisystem organ failure, COVID-19 infection, and yellow fever [1, 3, 6]. A unifying feature is the close association between these green inclusion bodies and lactic acidosis. Mortality has ranged from 31% in a review of 13 cases to 100% in a case series of six patients with COVID-19 [1, 2, 7]. Here we present a case of green inclusion bodies within neutrophils in a patient with aspiration pneumonia and severe pneumoperitoneum due to bowel perforation.

## Case presentation

**Figure 1:**
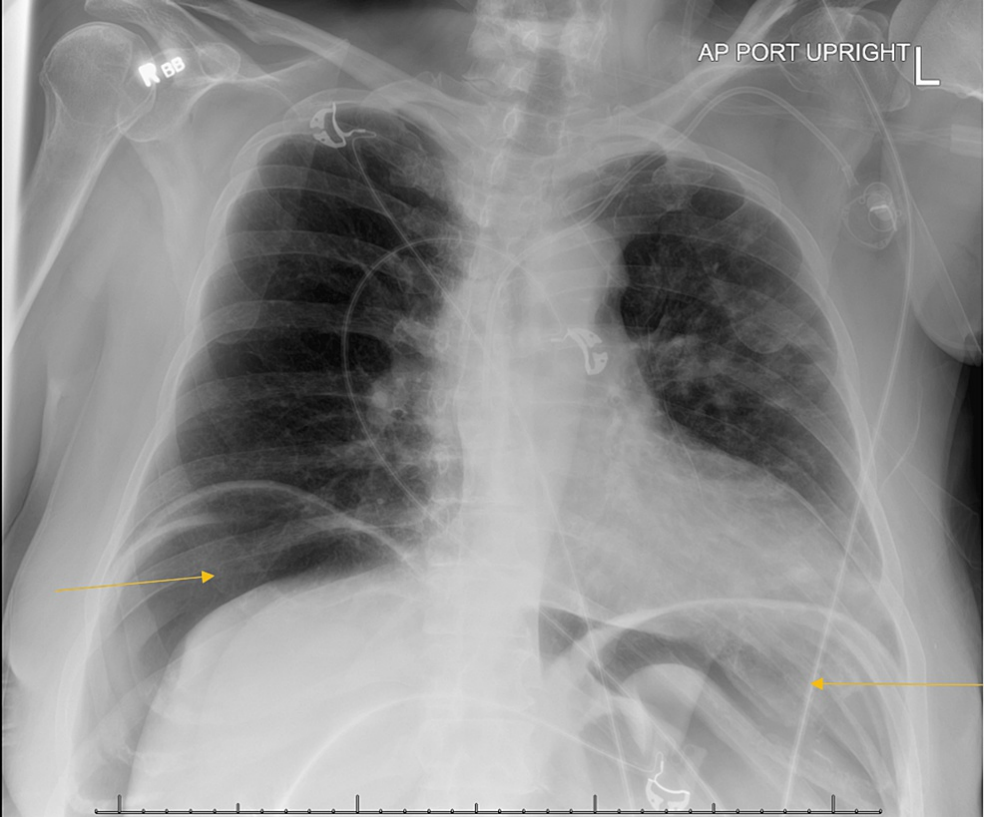
Chest X-ray performed after sigmoidoscopy demonstrating bilateral nodular infiltrates and intra-abdominal air (arrows).

The patient was a 63-year-old female with a history of invasive urothelial carcinoma (grade 4, stage II), transient ischemic attack, hypertension, and hyperlipidemia. She had previously undergone laparoscopic radical cystectomy with extracorporeal conduit creation four months prior to presentation. At the time of admission, she was receiving nivolumab immunotherapy to treat her urothelial carcinoma. She initially presented to the hospital from the clinic with nausea, vomiting, fatigue, and acute kidney injury (serum creatinine 2.5 mg/dL, baseline 0.9 mg/dL). Renal ultrasound revealed bilateral hydronephrosis, and she underwent bilateral nephrostomy tube placement. During this hospitalization, she developed an ileus and small bowel obstruction (SBO) presumed to be secondary to opioid-induced constipation. This was managed conservatively, and she was discharged home after a 15-day hospitalization. She returned to the hospital on the day of discharge due to persistent abdominal pain. At the time of readmission (hospital day #1), she continued complaining of nausea and vomiting but endorsed passing flatus. Her condition failed to improve, and a repeat CT scan of her abdomen obtained on hospital day #16 demonstrated partial obstructions of both the large and small bowel. In addition, a newly identified narrowing of the rectosigmoid junction was concerning for a possible mass. On hospital day #19, she was taken to the operating room for flexible sigmoidoscopy. Findings included extrinsic compression of the sigmoid colon with an inability to pass the scope beyond the compression. Post-procedure complications included witnessed aspiration and respiratory distress. Chest X-ray (Figure 1) demonstrated free air under the diaphragm and nodular opacities consistent with aspiration pneumonia/pneumonitis. The patient was intubated and transferred to the ICU for further management.

**Figure 2:**
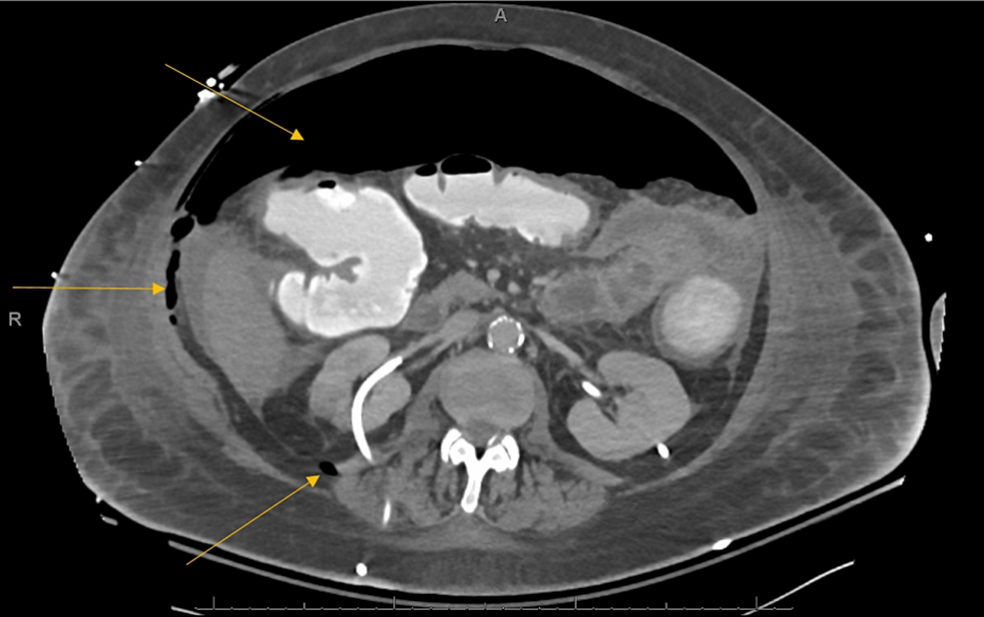
Diffuse-free intraperitoneal and retroperitoneal (arrows) air consistent with viscous perforation on CT scan.

**Figure 3:**
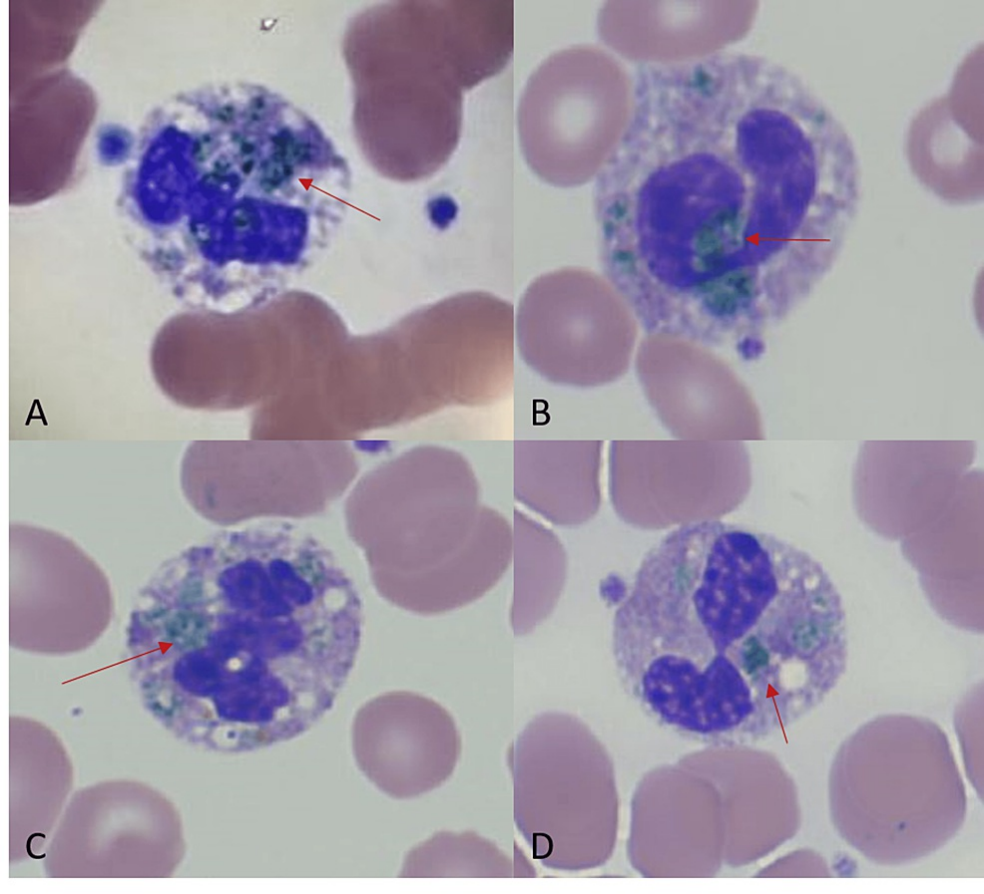
Blue-green cytoplasmic inclusion bodies (arrows) seen in four separate neutrophils (A-D) on hospital day #20.

Post-intubation bronchoscopy revealed copious thin brown secretions and bilaterally aspirated gastric contents throughout the lungs. She developed progressive shock despite administration of norepinephrine and vasopressin with notable worsening lactic acidosis to 9.7 mmol/L, acute renal failure with blood urea nitrogen (BUN) 39 mg/dL and creatinine 2.36 mg/dL, and acute hepatic injury with AST 1,275 U/L and ALT 710 U/L. Exploratory laparotomy was offered to the family and deferred due to the high risk of surgical mortality. Her abdomen's repeat CT scan (Figure 2) showed diffuse free intraperitoneal and retroperitoneal air concerning viscous perforation. On hospital day #20, a complete blood count was noted to have blue-green inclusion bodies within the neutrophils (Figure 3). After further discussion with the family, the decision was made to transition the patient to comfort measures only. The patient subsequently passed away. 

## Discussion

Blue-green inclusion bodies represent a rare but critical finding in patients with life-threatening illnesses. To the best of our knowledge, this is the first reported case of green inclusion bodies in a patient with complications of bowel perforation. The underlying etiology of these inclusion bodies remains unclear, but inclusion bodies were noted in our patient after developing septic shock requiring multiple vasopressors. The temporal association of these inclusion bodies with our patient's bowel perforation suggests that her acute intraabdominal infection and subsequent systemic inflammatory response syndrome may have contributed. Our patient rapidly decompensated and expired within 36 hours of the inclusion bodies being identified, highlighting their prognostic value in critically ill patients and the high risk of mortality once identified. A review of the current literature suggests a strong temporal association between the identification of the inclusion bodies and the subsequent onset of multisystem organ failure, irrespective of the patient's specific underlying illness [8-10]. Thus, once these inclusion bodies have been identified, this critical result should lead physicians to consider implementing more invasive vital sign monitoring, more frequent lab draws, and additional imaging to evaluate for worsening organ injury or failure. Previously published reports have projected that death will occur within 72 hours of identification of these inclusion bodies; however, our patient demonstrates that clinical deterioration can occur much more rapidly [4, 8].

While acute liver injury and elevated lactic acid have been closely linked to the development of these inclusion bodies, it remains to be determined whether these inclusion bodies result from hepatic ischemia in the setting of septic shock or direct hepatotoxicity [1, 3, 7, 9-11]. The specific mechanism by which these inclusion bodies develop and what material constitutes these inclusion bodies are unknown. However, the available pathology literature suggests these bodies are either lipofuscin or lipofuscin-like [2]. Lipofuscin is a product of oxidative stress on the cell that results from disturbances in proteostasis [12]. These disturbances in protein metabolism culminate in the formation of lipofuscin, an un-degradable hydrophobic protein aggregate [12]. While the blue-green inclusion bodies' staining pattern shares several similarities with lipofuscin, the staining pattern described is not totally consistent with previously described lipofuscin staining [2]. Analysis by Hodgson TO et al. [4] suggested that these inclusion bodies share some staining characteristics seen in sea-blue histiocytes that are typically seen in situations of rapid bone marrow cell death. These histologic findings of cellular stress correlate with the associated clinical severity and high mortality in patients with green neutrophilic inclusion bodies. While cell death and stress findings are not surprising in patients who have clinically decompensated and are hemodynamically unstable, laboratory evidence of these inclusion bodies could provide highly useful prognostic information for ICU patients. Strong consideration should be given to requiring laboratory staff to inform the clinical team of these findings in the same manner as any other critical lab value. The relative dearth of literature on this topic would also suggest that these "green crystals of death" are both under-recognized and underreported [13].

## Conclusions

The presence of green neutrophilic inclusion bodies in combination with acute liver injury and lactic acidosis carries a high risk of short-term mortality. The primary mechanism is unknown; however, hepatic ischemia and lactic acidosis are implicated. The role of pathophysiologic processes such as pneumoperitoneum, malignancy, and multi-organ failure (present in our patient) also cannot be excluded. Our patient's clinical course demonstrates that identifying these inclusion bodies can portend rapid decompensation and death. Physicians should be aware of the prognostic value of the presence of neutrophilic blue-green inclusion bodies and consider their presence as a "critical result," indicating the severity of their patient's illness.
